# MERS-coronavirus replication induces severe *in vitro* cytopathology and is strongly inhibited by cyclosporin A or interferon-α treatment

**DOI:** 10.1099/vir.0.052910-0

**Published:** 2013-08

**Authors:** Adriaan H. de Wilde, V. Stalin Raj, Diede Oudshoorn, Theo M. Bestebroer, Stefan van Nieuwkoop, Ronald W. A. L. Limpens, Clara C. Posthuma, Yvonne van der Meer, Montserrat Bárcena, Bart L. Haagmans, Eric J. Snijder, Bernadette G. van den Hoogen

**Affiliations:** 1 Molecular Virology Laboratory, Department of Medical Microbiology, Leiden University Medical Center, Leiden, The Netherlands; 2 Viroscience Lab, Erasmus MC, Rotterdam, The Netherlands; 3 Section Electron Microscopy, Department of Molecular Cell Biology, Leiden University Medical Center, Leiden, The Netherlands

## Abstract

Coronavirus (CoV) infections are commonly associated with respiratory and enteric disease in humans and animals. The 2003 outbreak of severe acute respiratory syndrome (SARS) highlighted the potentially lethal consequences of CoV-induced disease in humans. In 2012, a novel CoV (Middle East Respiratory Syndrome coronavirus; MERS-CoV) emerged, causing 49 human cases thus far, of which 23 had a fatal outcome. In this study, we characterized MERS-CoV replication and cytotoxicity in human and monkey cell lines. Electron microscopy of infected Vero cells revealed extensive membrane rearrangements, including the formation of double-membrane vesicles and convoluted membranes, which have been implicated previously in the RNA synthesis of SARS-CoV and other CoVs. Following infection, we observed rapidly increasing viral RNA synthesis and release of high titres of infectious progeny, followed by a pronounced cytopathology. These characteristics were used to develop an assay for antiviral compound screening in 96-well format, which was used to identify cyclosporin A as an inhibitor of MERS-CoV replication in cell culture. Furthermore, MERS-CoV was found to be 50–100 times more sensitive to alpha interferon (IFN-α) treatment than SARS-CoV, an observation that may have important implications for the treatment of MERS-CoV-infected patients. MERS-CoV infection did not prevent the IFN-induced nuclear translocation of phosphorylated STAT1, in contrast to infection with SARS-CoV where this block inhibits the expression of antiviral genes. These findings highlight relevant differences between these distantly related zoonotic CoVs in terms of their interaction with and evasion of the cellular innate immune response.

## Introduction

In June 2012, a previously unknown coronavirus was isolated from a 60-year-old Saudi Arabian patient who died from acute respiratory distress syndrome and multiple organ failure (Zaki *et al.*, 2012). Subsequently, the novel virus was isolated from several additional residents of and visitors to the Arabian Peninsula suffering from similar respiratory symptoms. In retrospect, a cluster of respiratory infections in Jordan (April 2012) was linked to the same agent, although no convincing evidence for human-to-human transmission was obtained. This was clearly different for a cluster of three UK cases in early 2013, consisting of a patient who had travelled to Saudi Arabia and two family members without recent travel history outside the UK. In the past year, various names have been used to refer to this newly identified CoV, including novel (beta)coronavirus (nCoV) and human coronavirus EMC (HCoV-EMC), but following a recent recommendation by the coronavirus study group of ICTV and other experts (de Groot *et al.*, 2013) we will use Middle East Respiratory Syndrome coronavirus (MERS-CoV) throughout this paper. Up to May 2013, 49 confirmed MERS cases, including 23 fatalities, have been recorded (http://www.who.int/csr/don/archive/disease/coronavirus_infections/en/).

Coronavirus (CoV) infections are associated with respiratory and enteric disease in humans and animals. Since the 1960s, two human CoVs (HCoVs OC43 and 229E) have been known to cause mild respiratory disease (Hamre & Procknow, 1966; McIntosh *et al.*, 1967), but it was the 2003 outbreak of severe acute respiratory syndrome (SARS; fatality rate ~10 %) that revealed the potentially lethal consequences of CoV-induced disease in humans (Drosten *et al.*, 2003; Ksiazek *et al.*, 2003). Two years later, bats were identified as the most likely animal reservoir for this zoonotic CoV (Lau *et al.*, 2005; Li *et al.*, 2005). Subsequently, a wide variety of bat-associated CoVs was discovered (Vijaykrishna *et al.*, 2007; Woo *et al.*, 2007), and two additional human CoVs (NL63 and HKU1) (Fouchier *et al.*, 2004; van der Hoek *et al.*, 2004; Woo *et al.*, 2005) were also identified. Although the general capacity of bat CoVs to switch hosts appears to be rather restricted (Müller *et al.*, 2012), the possibility of SARS-CoV re-emergence or zoonotic transfer of other animal CoVs has remained a public health concern over the past 10 years.

CoVs are classified in four genera (alpha-, beta-, gamma- and deltacoronaviruses; de Groot *et al.*, 2012), and our previous analysis of the MERS-CoV genome (van Boheemen *et al.*, 2012) identified the newly emerging agent as a member of lineage C of the genus *Betacoronavirus*. Strikingly, as in the case of SARS-CoV, the closest known relatives of MERS-CoV are bat coronaviruses, such as HKU-4 and HKU-5 (van Boheemen *et al.*, 2012; Woo *et al.*, 2007). The evolutionary distance to SARS-CoV (lineage B) is considerable, a notion further supported by recent comparative studies revealing important differences in receptor usage (Müller *et al.*, 2012; Raj *et al.*, 2013).

Mammalian viruses have to cope with the host cell’s innate responses, including those triggered by activation of the type I interferon (IFN) pathway (reviewed by Randall & Goodbourn, 2008). CoVs, including SARS-CoV, appear to have evolved a variety of mechanisms to block or evade such antiviral responses (reviewed by Perlman & Netland, 2009; Zhong *et al.*, 2012). For example, it was postulated that the sensing of dsRNA replication intermediates by the innate immune system is inhibited by the elaborate virus-induced membrane structures with which CoV RNA synthesis is associated (Knoops *et al.*, 2008; Versteeg *et al.*, 2007). Other evasion mechanisms have been attributed to protein functions that can be either conserved across CoVs or specific for certain CoV lineages. Proteins such as the non-structural protein nsp3 proteinase (Ratia *et al.*, 2006), the nsp16 2′-*O*-methyltransferase (Züst *et al.*, 2011), and the products of SARS-CoV ORFs 3b, 6 and 7a (Frieman *et al.*, 2007; Hussain *et al.*, 2008; Kopecky-Bromberg *et al.*, 2006; Zhou *et al.*, 2012), have all been described as preventing IFN induction/signalling. In particular, the SARS-CoV ORF6 protein is known to inhibit IFN-induced JAK-STAT signalling by blocking the nuclear translocation of phosphorylated STAT1 (p-STAT1), which contributes to the pathogenic potential of the virus in a mouse model (Sims *et al.*, 2013). In spite of these immune evasion strategies, treatment with type I IFNs can inhibit CoV replication *in vitro* (Garlinghouse *et al.*, 1984; Haagmans *et al.*, 2004; Paragas *et al.*, 2005; Taguchi & Siddell, 1985; Zheng *et al.*, 2004) and, for example, protects type I pneumocytes against SARS-CoV infection in macaques (Haagmans *et al.*, 2004).

Clearly, well-characterized systems for MERS-CoV replication in cell culture will be invaluable for future studies into basic virus properties and interactions with the host, including innate immune responses. Therefore, we set out to characterize the replication of MERS-CoV in different cell lines. Using this information, an assay to screen for antiviral compounds was developed, which identified cyclosporin A (CsA) as an inhibitor of MERS-CoV replication. Our first screening experiments also established that, compared with SARS-CoV, MERS-CoV replication is more sensitive to type I IFN treatment.

## Results

### Kinetics of MERS-CoV replication in Vero and Huh7 cells

Only a few laboratory studies on MERS-CoV replication have been reported thus far. Cells from a variety of mammalian hosts have been found to be susceptible, and infection can induce pronounced cytopathology and cell death (Müller *et al.*, 2012; Zaki *et al.*, 2012). Following entry, the CoV replicative cycle starts with translation of the positive-sense RNA genome into replicase polyproteins that are cleaved into 16 nsps (Gorbalenya *et al.*, 2006; van Boheemen *et al.*, 2012). These direct both genome replication and synthesis of the subgenomic (sg) mRNAs required to express the structural and accessory proteins. To investigate MERS-CoV replication in more detail, we used Vero and Huh7 cells to analyse viral RNA synthesis and progeny release in single-cycle infection experiments.

**Fig. 1.**
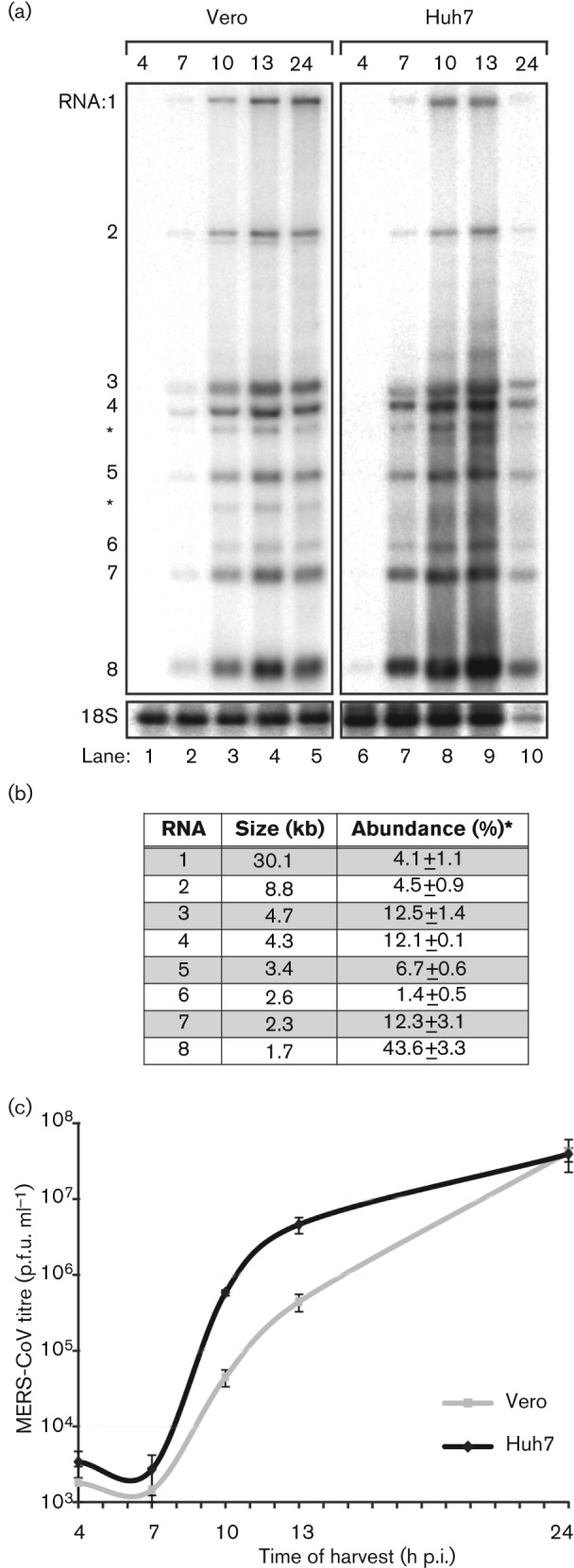
Kinetics of MERS-CoV replication in Vero and Huh7 cells. Vero and Huh7 cells were infected with MERS-CoV (m.o.i. of 5). (a) Hybridization analysis of viral mRNAs isolated from MERS-CoV-infected cells using an oligonucleotide recognizing the viral genome and all sg mRNAs. Additional minor bands of ~3 and ~4 kb were observed (*) and may represent additional viral mRNA species that remain to be studied in more detail. However, the corresponding positions in the ORF4a/b and ORF5 coding regions do not contain a canonical core TRS sequence (AACGAA; van Boheemen *et al.*, 2012) that might provide a direct explanation for their synthesis. (b) Analysis of the relative molarities of viral genome and each of the sg mRNAs (% of total viral mRNA). mRNA sizes were calculated on the basis of the TRS positions in the viral genome sequence (van Boheemen *et al.*, 2012). Phosphorimager quantification was performed on the gel lanes with the RNA samples isolated from Vero cells at 10, 13 and 24 h p.i. (Fig. 1a; lanes 3–5, respectively; mean±sd). (c) Release of infectious MERS-CoV progeny into the medium of infected Vero or Huh7 cells at the indicated time points, as determined by plaque assay (mean±sd; *n* = 4).

Hybridization analysis of the accumulation of viral RNA revealed the presence of genome RNA and seven sg transcripts, with sizes closely matching those predicted previously from the positions of conserved transcription regulatory sequences (TRSs) in the viral genome (van Boheemen *et al.*, 2012) (Fig. 1a). The relative abundance of the various sg mRNAs was similar to what has been observed for other CoVs, with the smallest species, encoding the nucleocapsid (N) protein, being by far the most abundant transcript (Fig. 1b). In both cell lines, viral mRNAs could be readily detected at 7 h post-infection (p.i.) and reached maximum levels around 13 h p.i. (Fig. 1a). Viral RNA levels remained more or less constant until 24 h p.i. in Vero cells, whereas the amount isolated from Huh7 cells declined due to the more rapid development of cytopathology in this cell line between 13 and 24 h p.i. (see below). After the peak of viral RNA accumulation had been reached, the titre of virus released from MERS-CoV-infected Vero cells increased steadily from ~5×10^5^ to ~5×10^7^ p.f.u. ml^−1^ (Fig. 1c). Interestingly, the bulk of the viral progeny was released significantly earlier from Huh7 cells, although the final titre at 24 h p.i. was comparable to that obtained from Vero cells.

### Antisera raised against non-structural proteins of other betacoronaviruses cross-react with MERS-CoV proteins

**Fig. 2.**
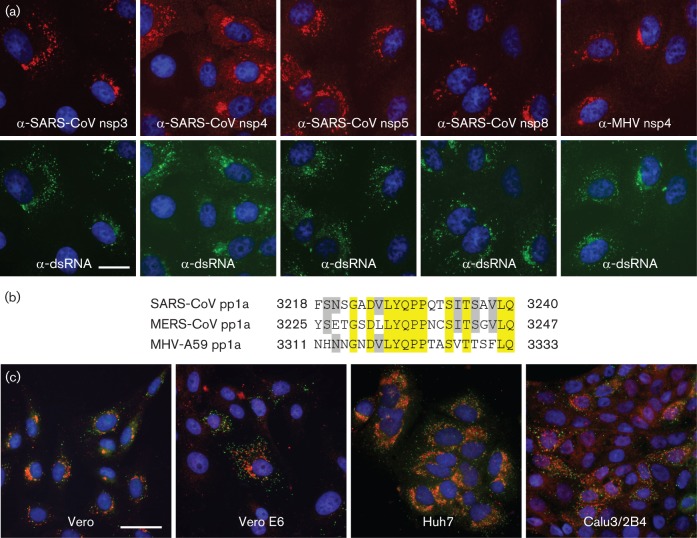
Selected rabbit antisera raised against SARS-CoV and mouse hepatitis virus (MHV) nsps cross-react with MERS-CoV proteins. (a) MERS-CoV-infected Vero cells (m.o.i. of 5) were fixed at 8 h p.i. For immunofluorescence microscopy, cells were double-labelled with a mouse mAb recognizing dsRNA (bottom row) and rabbit antisera raised against SARS-CoV nsp3, nsp4, nsp5 or nsp8, or MHV nsp4 (top row). Bar, 20 µm. (b) Sequence comparison of the C-terminal domain of nsp4 of SARS-CoV (isolate Frankfurt 1), MERS-CoV (strain EMC/2012) and MHV (strain A59). The SARS-CoV and MHV sequences correspond to the synthetic peptides used to raise rabbit anti-nsp4 sera. Residues conserved in all three viruses are highlighted in yellow, whereas residues conserved in two out of three are highlighted in grey. Amino acid numbers refer to the full-length pp1a sequence. (c) Monolayers of Vero, Vero E6, Huh7 and Calu3/2B4 cells were infected with MERS-CoV (m.o.i. of 5) and double-labelled for dsRNA (green) and nsp3 (red). Bar, 40 µm.

Despite the relatively large evolutionary distance to better-characterized CoVs, we tested a panel of antisera from our laboratory for cross-reactivity with MERS-CoV-infected cells. In contrast to a polyclonal serum recognizing the SARS-CoV N protein (data not shown), antisera against various SARS-CoV nsps (nsp3, nsp5 and nsp8; Snijder *et al.*, 2006) raised using purified recombinant proteins as antigen were found to cross-react strongly (Fig. 2a). In addition, rabbit antisera raised against synthetic peptides (23mers) representing a small but conserved C-terminal part of SARS-CoV and MHV nsp4 strongly cross-reacted with MERS-CoV. Only small but apparently immunogenic parts of these peptides (e.g. LYQPP) are absolutely conserved between MHV and MERS-CoV nsp4 (Fig. 2b). Conservation in other betacoronaviruses (data not shown) suggests that antisera recognizing this nsp4 region may be used for immunodetection of additional (newly emerging) CoVs.

### MERS-CoV replication structures

Subsequently, we employed a mAb recognizing dsRNA to localize intermediates in viral RNA synthesis (Knoops *et al.*, 2008; Weber *et al.*, 2006). In various cell types, the immunolabelling signals for both replicase and dsRNA localized to the perinuclear region (Fig. 2c), where the replication structures induced by other CoVs are known to accumulate (Brockway *et al.*, 2003; Gosert *et al.*, 2002; Knoops *et al.*, 2008; Snijder *et al.*, 2006; Stertz *et al.*, 2007; Ulasli *et al.*, 2010).

**Fig. 3.**
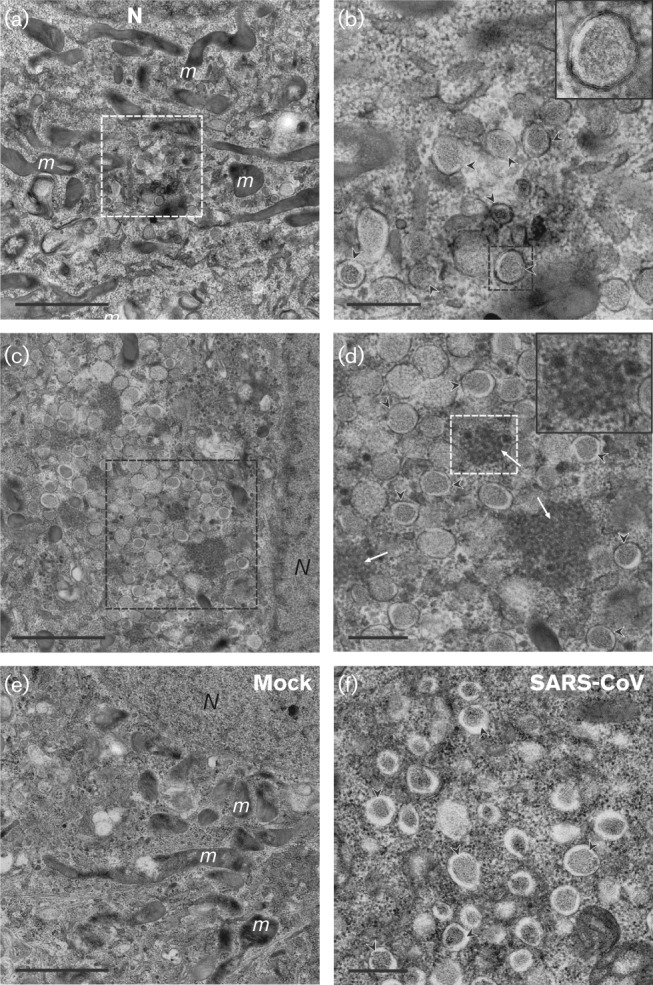
Membrane structures induced by MERS-CoV infection. (a–d) Electron micrographs of thin sections (100 nm) of MERS-CoV-infected Vero cells at 8 h p.i. Low magnification images of a cell containing a small cluster of DMVs (a), enlarged in (b). Some DMVs are indicated by black arrowheads and the inset displays a higher magnification of the boxed DMV in (b). Extensive membrane alterations in the perinuclear region are shown in (c), with the boxed area displayed at higher magnification in (d), where CMs (white arrows, inset) embedded in clusters of DMVs (black arrowheads) can be observed. (e, f) For comparison, (e) shows the unaltered cytoplasm of a mock-infected cell and (f) contains SARS-CoV-induced DMV (black arrowheads) as observed after HPF and freeze substitution. N, nucleus; m, mitochondria. Bars, 2 µm (a, c, e); 500 nm (b, d, f).

We next used EM to investigate the ultrastructural and potentially cytopathic changes that MERS-CoV induces in infected cells, and focused on the membranous replication structures that support MERS-CoV RNA synthesis. The preservation of such structures, typically double-membrane vesicles (DMVs) and convoluted membranes (CMs), was found previously to be improved significantly by using protocols that include cryofixation and freeze substitution (Knoops *et al.*, 2008; Snijder *et al.*, 2006). Therefore, we applied these advanced preservation techniques, including newly developed protocols for high-pressure freezing (HPF), to MERS-CoV-infected Vero cells. Images of similarly prepared SARS-CoV-infected Vero E6 cells were included for comparison (Fig. 3f).

Compared with mock-infected control cells (Fig. 3e), different degrees of distinct alterations were observed at 8 h p.i. Some cells contained relatively small DMV clusters (Fig. 3a, b; black arrowheads and inset), whereas in others large numbers of DMVs occupied extensive areas of the perinuclear region (Fig. 3c, d), differences that probably reflect different stages in infection progression. The diameter of MERS-CoV-induced DMVs ranged from 150 to 320 nm, comparable to what was measured previously for SARS-CoV-induced structures (Knoops *et al.*, 2008). An interesting morphological difference with our previous studies of SARS-CoV-infected cells was the presence of a dense inner DMV core, which can be attributed to technical differences in sample preparation. In terms of ultrastructural preservation, HPF is widely considered superior to the previously used plunge-freezing protocols. Also, in the case of SARS-CoV (Fig. 3f) and the distantly related equine arteritis virus (Knoops *et al.*, 2012), a similar dense DMV core became apparent when HPF was employed. Although DMV cores are known to contain dsRNA, the implications of these ultrastructural observations remain unclear. Interestingly, CMs were always surrounded by DMV clusters and were only observed in cells that appeared to be more advanced in infection (Fig. 3c, d; white arrows and inset). This observation strengthens the notion that DMV formation precedes the development of CMs, as postulated previously for SARS-CoV (Knoops *et al.*, 2008).

### MERS-CoV-induced cytophatology and cell death

**Fig. 4.**
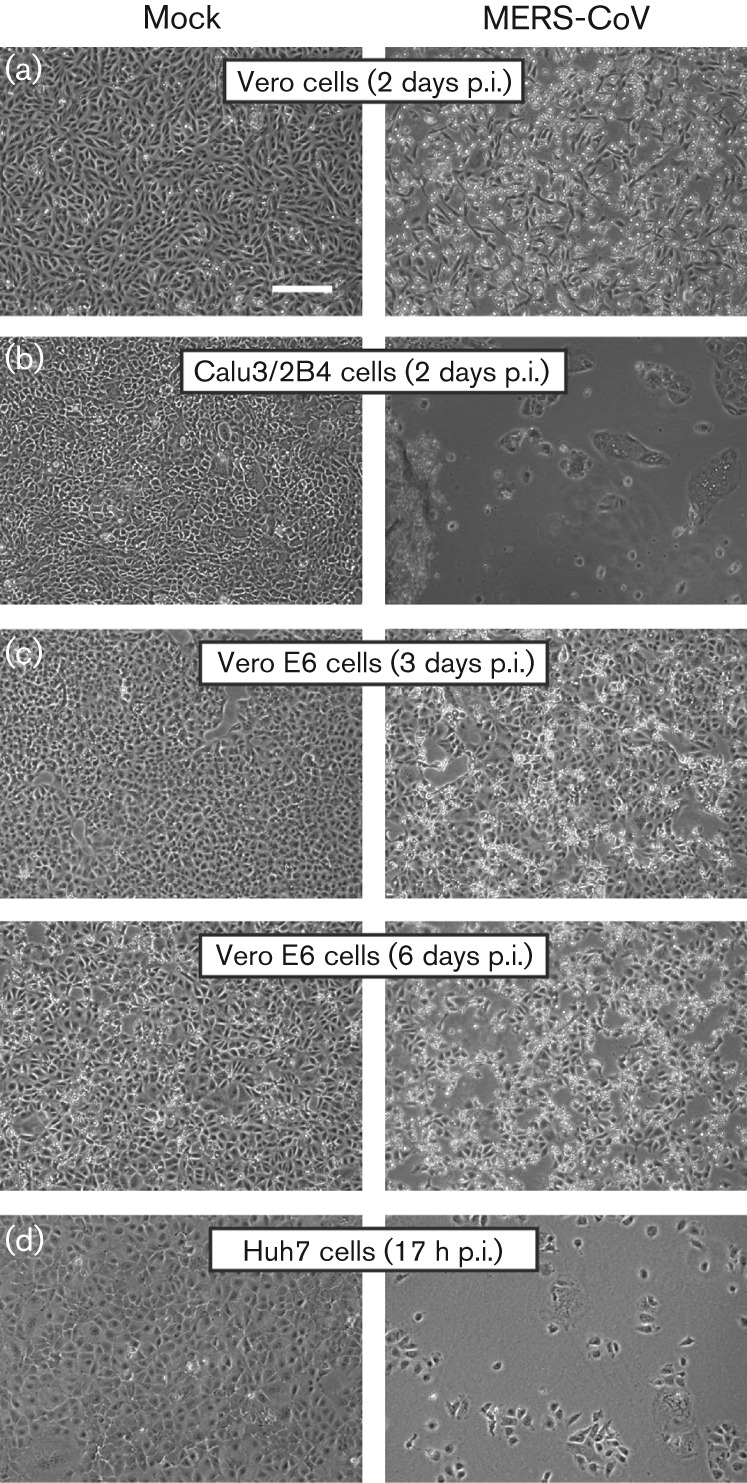
MERS-CoV infection induces severe cytopathology in monkey and human cell lines. Monolayers of Vero (a), Calu3/2B4 (b), Vero E6 (c) and Huh7 (d) cells were infected with MERS-CoV (m.o.i. of 0.05) and analysed by light microscopy at the indicated time points. Bar, 100 µm.

In cell culture, many CoVs induce severe cytopathic effect (CPE) and cell death. Infection with a number of CoVs can also induce extensive syncytium formation, due to fusion activity of the viral spike protein at neutral pH (reviewed by Belouzard *et al.*, 2012). MERS-CoV-induced cytopathology was monitored by light microscopy following low m.o.i. inoculation of monkey and human cells (Fig. 4). In line with previous observations (Zaki *et al.*, 2012), Vero cells developed clear CPE at 2 days p.i. and detached at 3 days p.i. (Fig. 4a). Similar observations were made for Calu3/2B4 cells (Fig. 4b). In contrast, MERS-CoV-infected Vero E6 cells displayed only mild CPE starting at 3 days p.i and cell death was not complete after 6 days (Fig. 4c). The development of CPE in Huh7 cells was strikingly faster compared with the three other cell lines and, following extensive syncytium formation, cells had already detached by around 17 h (Fig. 4d). Given the low m.o.i. used and the virus replication kinetics (Fig. 1), the syncytium formation in these only partially infected Huh7 cultures appeared to be a major factor in CPE development. Dipeptidyl peptidase-4 (DPP4) expression on Vero and Huh7 cells (Raj *et al.*, 2013) and expression levels of DPP4 on Calu3/2B4 and Vero E6 cells correlated with susceptibility to MERS-CoV (data not shown).

### Development of an assay to screen for compounds inhibiting MERS-CoV replication

**Fig. 5.**
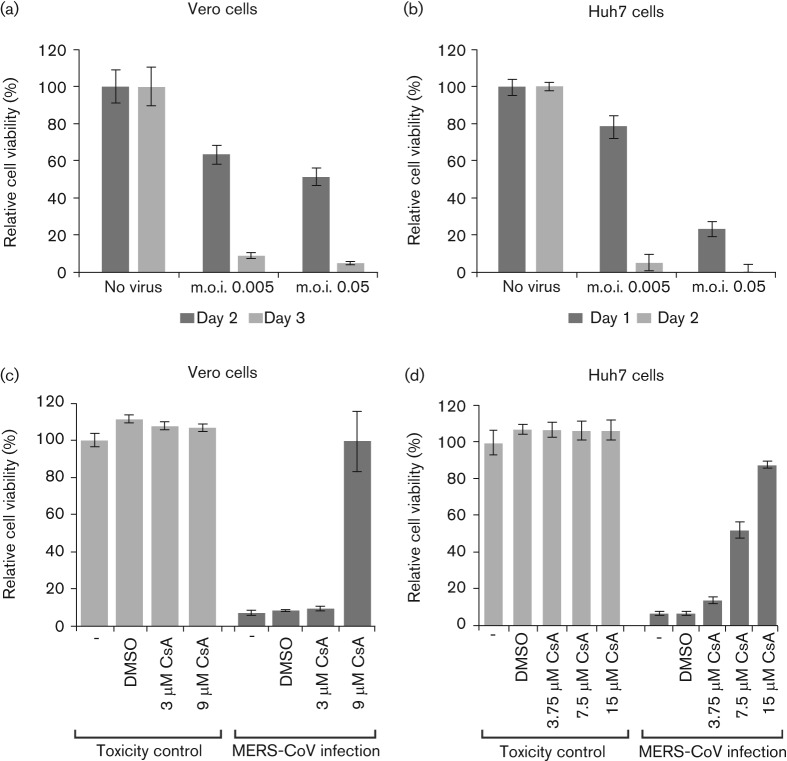
Development of an assay to screen for compounds inhibiting MERS-CoV replication. Vero (a, c) and Huh7 (b, d) cells in a 96-well plate format were infected at an m.o.i. of 0.005 or 0.05. Mock-infected cells (no virus) were used as a reference for unchanged cell viability (their relative viability was set at 100 %). Infected Vero cells were incubated for 2 (dark shading) or 3 (light shading) days (a) and Huh7 cells were incubated for 1 (dark shading) or 2 (light shading) days (b). (c) Vero cells were infected (dark shading) or not (light shading) with MERS-CoV (m.o.i. of 0.005) in the presence of 3 or 9 µM CsA, or 0.09 % DMSO as a solvent control. (d) Huh7 cells were infected (dark shading) or not (light shading) with MERS-CoV (m.o.i. of 0.005) in the presence of 3.75, 7.5 or 15 µM CsA, or 0.15 % DMSO. The graphs in (c) and (d) show the results of a representative experiment (mean±sd; *n* = 4). All experiments were repeated at least twice.

The virus-induced CPE in Vero and Huh7 cells was used to develop a first assay to screen for compounds that inhibit MERS-CoV replication in cell culture. Vero cells were seeded in 96-well plates and infected at an m.o.i. of 0.005 or 0.05 (Fig. 5a). After 2 and 3 days, CPE formation was monitored microscopically and cytotoxicity was measured using a commercial cell viability assay. Moderate CPE was observed on day 2, and by day 3 cell viability in uninfected cells had dropped below 10 % with both virus doses used (Fig. 5a), indicating near-complete cell death. In MERS-CoV-infected Huh7 cells (Fig. 5b), by day 1, cell viability had dropped to 79 or 24 % (after m.o.i. 0.005 or 0.05 infection, respectively), which was in line with our observations on rapid syncytium formation and CPE in this particular cell line (Fig. 4d). By day 2, CPE was complete for both virus doses used and cells had detached (Fig. 5b). Based on this comparison, further experiments were carried out using an m.o.i. of 0.005, and Huh7 and Vero cells were incubated for 2 or 3 days, respectively, before measuring cell viability.

Previously, it was shown that replication of various CoVs, including SARS-CoV, can be inhibited by the immunosuppressive drug CsA (de Wilde *et al.*, 2011; Pfefferle *et al.*, 2011). Therefore, whilst testing whether the CPE-based assay described above could be used as an antiviral screening method, we used CsA treatment to obtain a first proof of principle. Infected Vero cells were treated with 3 or 9 µM CsA and analysed at 3 days p.i. At the concentrations used, CsA did not adversely affect the viability of mock-infected cells (Fig. 5c). Treatment with 9 µM completely inhibited CPE and left cell viability unchanged compared with mock-infected control cells. The inhibitory effect of CsA was confirmed in Huh7 cells (Fig. 5d), which displayed a reduced or lack of CPE upon treatment with 7.5 and 15 µM CsA, respectively. These results were corroborated by immunofluorescence microscopy analysis of CsA-treated and high m.o.i.-infected Vero and Huh7 cells, and by determining virus titres released into the medium. Both assays confirmed an almost complete block of MERS-CoV infection (data not shown). However, as reported previously for other CoVs (de Wilde *et al.*, 2011), a small fraction of MERS-CoV-infected cells appeared to be refractive to CsA treatment and supported a low level of MERS-CoV replication, even at high CsA concentrations (data not shown).

### Enhanced sensitivity of MERS-CoV to pegylated IFN-α treatment in comparison with SARS-CoV

Type I IFNs inhibit CoV replication and can protect against infection in animal models (Haagmans *et al.*, 2004; Taguchi & Siddell, 1985). We therefore compared the effect of pegylated IFN-α (PEG-IFN) treatment on MERS-CoV and SARS-CoV replication *in vitro*. Vero cells were given PEG-IFN 4 h before low-m.o.i. infection, together with the inoculum, or 4 h after infection. At 2 days p.i., CPE was scored microscopically.

**Fig. 6.**
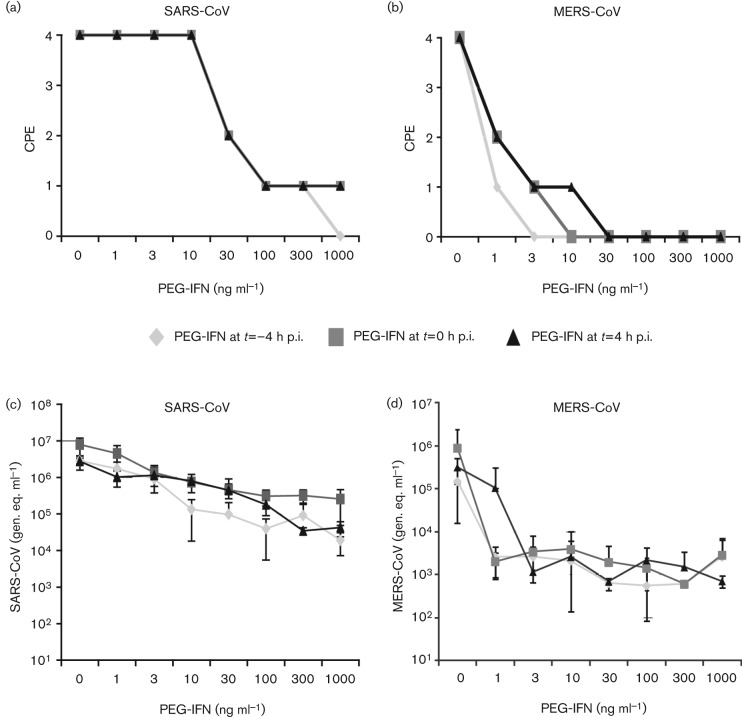
Sensitivity of MERS-CoV and SARS-CoV to PEG-IFN. Vero cells were incubated with 0–1000 ng PEG-IFN ml^−1^ at *t* = −4, *t* = 0 and *t* = 4 h p.i. Cells were infected with 100 TCID_50_ virus per well (a, b). At 2 days p.i., cells were examined for CPE. The effect of PEG-IFN treatment on CPE induced by SARS-CoV (a) or MERS-CoV (b) is shown. CPE was scored as none (0), mild (1), moderate (2), severe (3) or complete (4). (c, d) Viral genomes in the culture medium of virus-infected cells were determined by RT-PCR. The influence of PEG-IFN treatment on the viral RNA load [genome equivalents (gen. eq.) ml^−1^] in the supernatants of cells infected with SARS-CoV (c) or MERS-CoV (d) is shown.

Treatment with PEG-IFN profoundly inhibited both MERS-CoV- and SARS-CoV-induced CPE and RNA levels in a dose-dependent manner (Fig. 6). At 2 days p.i., SARS-CoV-induced CPE was reduced for all time points of PEG-IFN addition when using a dose of at least 30 ng PEG-IFN ml^−1^ (Fig. 6a), whereas MERS-CoV-induced CPE had already decreased using a dose of 1 ng ml^−1^ (Fig. 6b). For SARS-CoV, only pre-treatment with 1000 ng PEG-IFN ml^−1^ completely prevented CPE. For MERS-CoV, complete inhibition of CPE was observed at much lower concentrations, specifically 3, 10 or 30 ng ml^−1^, when the drug was added to the cells before, during or after infection, respectively. Although decreased CPE was also observed in SARS-CoV-infected cultures treated with 30 ng PEG-IFN ml^−1^, only a 30-fold reduction in viral RNA was detected in their medium at 2 days p.i. (Fig. 6c). For comparison, treatment of MERS-CoV-infected cells with the same PEG-IFN dose completely blocked CPE and reduced viral RNA levels in the medium by 600- to 2000-fold, depending on the timing of PEG-IFN addition (Fig. 6d).

**Fig. 7.**
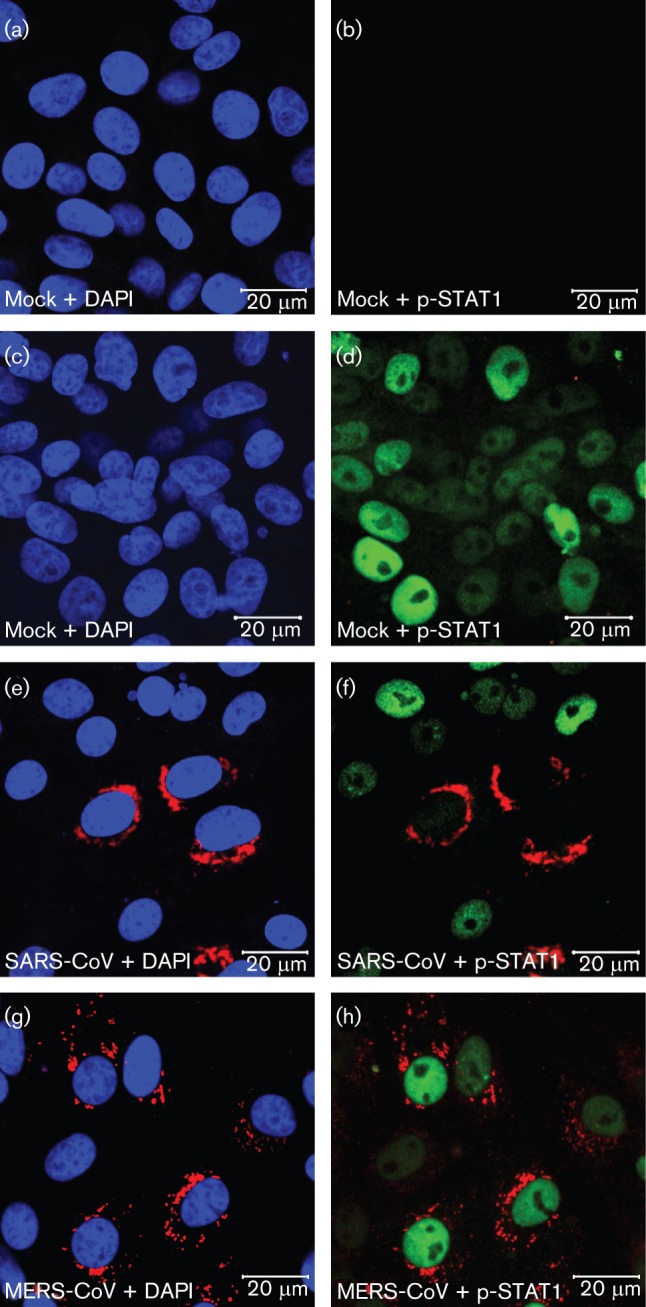
IFN-α induced nuclear translocation of p-STAT1 in MERS-CoV-infected Vero cells. Confocal immunofluorescence microscopy of uninfected Vero cells (a–d) and Vero cells infected (m.o.i. of 1) with SARS-CoV (e, f) or MERS-CoV (g, h). At 8 h p.i. cells were left untreated (a, b) or treated (c–h) with 1000 ng PEG-IFN ml^−1^ for 30 min, fixed and double-labelled with antisera against SARS-CoV nsp3 (red; a–h), or p-STAT1 (green; b, d, f, h), and nuclear DNA was stained with DAPI (blue; a, c, e, g).

Our data revealed that, in the same cell line, MERS-CoV infection was 50–100 times more sensitive to PEG-IFN treatment than SARS-CoV infection. This difference may be explained by important lineage-specific genetic differences between these two zoonotic betacoronaviruses in terms of accessory protein genes encoded in the 3′ part of the genome (Snijder *et al.*, 2003; van Boheemen *et al.*, 2012). In particular, MERS-CoV does not encode a homologue of the SARS-CoV ORF6 protein, which was reported to block the IFN-induced nuclear translocation of phosphorylated transcription factor STAT1. As nuclear translocation of p-STAT1 is essential for transcriptional activation of downstream antiviral genes, the ORF6 protein makes SARS-CoV less sensitive to treatment with type I IFN (Frieman *et al.*, 2007; Sims *et al.*, 2013). IFN-induced translocation of p-STAT1 was readily observed in IFN-treated mock-infected Vero cells (Fig. 7a–d), but not in IFN-treated SARS-CoV-infected cells (Fig. 7e, f). In contrast, in MERS-CoV-infected and IFN-treated cultures, the translocation of p-STAT1 was detected (Fig. 7 g, h). Together with the data on IFN sensitivity (Fig. 5), these observations highlight important differences between SARS-CoV and MERS-CoV in terms of their interaction with the IFN signalling pathways.

## Discussion

Following the 2003 SARS epidemic, global CoV hunting efforts identified a wealth of previously unknown family members, in particular in bat species from several continents (de Groot *et al.*, 2012). Moreover, at least three of the four current ‘established’ human CoVs (NL63, 229E and OC43) were postulated to have originated from zoonotic reservoirs (Huynh *et al.*, 2012; Pfefferle *et al.*, 2009; Vijgen *et al.*, 2005). Recently, about a decade after the SARS outbreak, MERS-CoV was identified as the next zoonotic CoV (Zaki *et al.*, 2012) and appears to be highly pathogenic to humans: of the 49 cases confirmed thus far, 23 had a fatal outcome (http://www.who.int/csr/don/archive/disease/coronavirus_infections/en/). Whether zoonotic CoVs cause transient epidemics or establish a long-lasting relationship with the human host, an in-depth understanding of virus–host interactions will be required to develop effective countermeasures. In this study, we defined several basic but important parameters of MERS-CoV replication in cell culture (Figs 1–4). Among the tools for MERS-CoV research developed are immunoassays based on cross-reacting antisera raised against other betacoronaviruses (Fig. 2) and a CPE-based assay that can be used to screen for antiviral effects (Figs 5 and 6).

Following the development of a high-throughput screening method for antiviral effects, proof of principle was obtained using CsA, a recently discovered inhibitor of CoV replication (de Wilde *et al.*, 2011; Pfefferle *et al.*, 2011). This drug affects the function of several members of the cellular cyclophilin family and appears to block functional interactions between viral proteins and one or multiple cyclophilin family members (Nagy *et al.*, 2011). Low-micromolar CsA concentrations blocked MERS-CoV-induced CPE in Vero and Huh7 cells (9 and 15 µM, respectively), as observed previously for other CoVs (de Wilde *et al.*, 2011; Pfefferle *et al.*, 2011). As in these previous studies (de Wilde *et al.*, 2011), a small fraction of the cells somehow remained susceptible to MERS-CoV infection, even at high CsA concentrations. Thus, virus replication could not be completely eliminated, which may ultimately lead to the development of CsA resistance in cell culture. In conclusion, these experiments established that monitoring MERS-CoV-induced CPE could be a valuable and rapid tool in screening for the potential antiviral activity of, for example, small-molecule compounds or Food and Drug Administration-approved drugs such as PEG-IFN.

Type I IFN induction, a hallmark of the early innate immune response, is counteracted by different CoV-encoded proteins. Despite these evasion strategies, IFN can be detected in sera of CoV-infected mice and humans (Cameron *et al.*, 2012; Garlinghouse *et al.*, 1984; Taguchi & Siddell, 1985), and CoV-infected plasmacytoid dendritic cells have been identified as a source of high IFN-α levels (Cervantes-Barragan *et al.*, 2007; Roth-Cross *et al.*, 2007). The SARS-CoV ORF6 protein, however, (partially) disrupts the downstream IFN-induced signalling in infected cells by inhibiting the nuclear translocation of p-STAT1, a critical component of both the IFN-α and IFN-γ signalling pathways (Frieman *et al.*, 2007). Although contributions from additional immune evasion mechanisms are likely, the lack of a SARS-CoV ORF6 homologue (van Boheemen *et al.*, 2012) may be a major factor in the higher sensitivity of MERS-CoV to PEG-IFN treatment, as observed in this study and other recent work (Kindler *et al.*, 2013). This was substantiated further by the finding that nuclear translocation of p-STAT1 is not blocked in MERS-CoV-infected cells (Fig. 7), which indicates that MERS-CoV has not evolved an alternative strategy to achieve the same goal. MHV has been shown to be relatively insensitive to IFN pre-treatment; however, this virus also does not block activation and translocation of p-STAT1 but instead inhibits the induction of a subset of ISGs by IFN-α/β (Rose *et al.*, 2010). Future studies may elucidate whether MERS-CoV has evolved alternative strategies to cope with the host’s IFN response. In addition, it will be important to test whether MERS-CoV is attenuated *in vivo* as a result of the relative high IFN sensitivity.

PEG-IFN is a registered drug used for the treatment of chronic hepatitis B and C infections in humans (Bergman *et al.*, 2011). Several CoVs, including SARS-CoV, have been shown to be sensitive to both type I IFN treatment *in vitro* and PEG-IFN treatment *in vivo* (Haagmans *et al.*, 2004; Paragas *et al.*, 2005; Zheng *et al.*, 2004), and in this study we established a relatively high sensitivity for MERS-CoV. For example, in cynomolgus macaques, plasma levels of 1–5 ng ml^−1^ were reached (Haagmans *et al.*, 2004), a dose that in this study significantly reduced MERS-CoV replication *in vitro*. The sensitivity of MERS-CoV to exogenous IFN suggests that administration of recombinant IFN merits further evaluation as a therapeutic intervention strategy if new infections with this novel virus occur.

## Methods

### 

#### Cells culture and virus infection.

Vero cells (ATCC CCL-81) were cultured in Eagle’s minimal essential medium (EMEM; Lonza) with 8 % FCS (PAA) and antibiotics. Huh7 cells were grown in Dulbecco’s modified Eagle’s medium (DMEM; Lonza) containing 8 % FCS, 2 mM l-glutamine (PAA), non-essential amino acids (PAA) and antibiotics. Vero E6 and Calu3/2B4 cells were cultured as described previously (Snijder *et al.*, 2006; Yoshikawa *et al.*, 2010). Infection of Vero, Vero E6, Huh7 and Calu3/2B4 cells with MERS-CoV (strain EMC/2012; Zaki *et al.*, 2012; van Boheemen *et al.*, 2012) at high m.o.i. (m.o.i. of 5) was carried out in PBS containing 50 µg DEAE-dextran ml^−1^ and 2 % FCS. Inoculations with a low dose (m.o.i. ≤ 0.05) of MERS-CoV or SARS-CoV (strain HKU-39849; Zeng *et al.*, 2003) were carried out directly in EMEM containing 2 % FCS. Virus titrations by plaque assay were performed as described previously (van den Worm *et al.*, 2012). All work with live MERS-CoV and SARS-CoV was performed inside biosafety cabinets in Biosafety Level 3 facilities at Leiden University Medical Center or Erasmus Medical Center.

#### Antibodies and drugs.

Rabbit antisera recognizing the SARS-CoV replicase subunits nsp3, nsp4, nsp5 and nsp8 have been described previously (Snijder *et al.*, 2006; van Hemert *et al.*, 2008b). Rabbit antisera recognizing the SARS-CoV nucleocapsid (N) protein and MHV nsp4 were raised as described elsewhere (Snijder *et al.*, 1994). Antigens were a full-length recombinant SARS-CoV N protein (purified from *Escherichia coli*) and a synthetic peptide representing the 23 C-terminal residues of MHV nsp4, respectively. p-STAT1 was detected with Alexa Fluor 488-labelled mouse anti-STAT1 (pY701; BD Biosciences), and FITC-labelled anti-mouse IgG was used to enhance the green fluorescence. Virus infection was detected using the above-mentioned anti-nsp3 sera and Alexa Fluor 594-labelled anti-rabbit IgG.

CsA (Sigma) was dissolved in DMSO and a 10 mM stock was stored in aliquots for single use at −20 °C. Peg-interferon α-2b (PEG-IFN; Pegintron) was prepared according to the manufacturer’s instruction as a 100 µg ml^−1^ stock and stored at 4 °C.

#### Immunofluorescence microscopy.

Cells were grown on coverslips and fixed with 3 % paraformaldehyde in PBS or with 4 % formaldehyde and 70 % ethanol (p-STAT1 experiments), permeabilized with 0.1 % Triton X-100 and processed for immunofluorescence microscopy as described previously (van der Meer *et al.*, 1998). Specimens were examined with a Zeiss Axioskop 2 fluorescence microscope with an Axiocam HRc camera and Zeiss Axiovision 4.4 software or with a confocal microscope (Zeiss, LSM 700) (p-STAT1 experiments).

#### EM.

Vero cells were grown on sapphire discs and fixed at 8 h p.i. for 30 min at room temperature with 3 % paraformaldehyde and 0.25 % glutaraldehyde in 0.1 M PHEM buffer pH 6.9 [60 mM piperazide-1,4-bis (2-ethanesulfonic acid), 25 mM HEPES, 2 mM MgCl_2_, 10 mM EGTA] containing 50 % diluted EMEM and 1 % FCS. Cells were stored in fixative at 4 °C for 72 h and then high-pressure frozen using a Leica EM PACT2. Freeze substitution was performed in an automated system (Leica AFS2) using as freeze-substitution medium acetone containing 1 % OsO_4_, 0.5 % uranyl acetate and 10 % H_2_O. First, the samples were maintained at −90 °C for 6 h in this medium and then slowly warmed to −20 °C within 14 h, kept at −20 °C for 1 h, warmed to 0 °C at a 5 °C h^−1^ rate and left at 0 °C for 1 h before letting the samples reach room temperature. After washing with acetone, the samples were gradually infiltrated with epoxy resin LX-112 and polymerized at 60 °C. The samples were cut into thin sections (100 nm) and counterstained with uranyl acetate and lead citrate. Imaging was performed in an FEI Tecnai12 TWIN electron microscope operating at 120 kV and equipped with an Eagle 4k cooled slow-scan charge-coupled device camera (FEI Company). The images were acquired using binning mode 2.

#### Intracellular viral RNA analysis.

Isolation of intracellular viral RNA was carried out as described previously (van Kasteren *et al.*, 2013). After drying of the gel, viral mRNAs were detected by hybridization with a ^32^P-labelled oligonucleotide probe (5′-GCAAATCATCTAATTAGCCTAATC-3′) complementary to the 3′ end of all MERS-CoV mRNAs. Equal loading was verified in a second hybridization using a ^32^P-labelled oligonucleotide probe (5′-GTAACCCGTTGAACCCCATT-3′) recognizing 18S rRNA (van Hemert *et al.*, 2008a). ImageQuant TL software (GE Healthcare) was used for quantification.

#### Real-time reverse transcription-PCR (RT-PCR).

RNA from 200 µl culture medium of CoV-infected cells was isolated with a MagnaPure LC Total Nucleic Acid Isolation kit (Roche) and eluted in 100 µl. RT-PCR conditions for quantifying MERS-CoV and SARS-CoV RNA and amplification parameters have been described previously (Kuiken *et al.*, 2003; Raj *et al.*, 2013). Dilutions of viral RNA isolated from MERS-CoV or SARS-CoV virus stocks with a known virus titre were used to produce a standard curve.

#### Development of a screening assay for antiviral compounds.

Huh7 or Vero cells were seeded in 96-well plates at a density of 1×10^4^ or 2×10^4^ cells per well, respectively. After overnight growth, cells were infected with an m.o.i. of 0.005 or 0.05. At 1–3 days after incubation, differences in cell viability caused by virus-induced CPE or by compound-specific side effects were analysed using the CellTiter 96 AQ_ueous_ Non-Radioactive Cell Proliferation Assay (Promega), according to the manufacturer’s instructions. Absorbance at 490 nm (*A*_490_) was measured using a Berthold Mithras LB 940 96-well plate reader. Infected cells were given CsA or DMSO (solvent control) prior to infection (m.o.i 0.005). Cytotoxic effects caused by CsA treatment alone were monitored in parallel plates containing mock-infected cells.

#### IFN sensitivity and p-STAT1 translocation experiments.

One day prior to infection, Vero cells were plated at a density of 10^4^ cells per well in a 96-well plate format. At −4, 0 and 4 h p.i., cells were incubated with 0–1000 ng PEG-IFN ml^−1^ in 250 µl. At *t* = 0 h, all wells were washed with PBS and infected with MERS-CoV or SARS-CoV (100 TCID_50_ per 100 µl medium). Those cultures receiving treatment from *t* = −4 or *t* = 0 were infected in the presence of the indicated concentration PEG-IFN. After 1 h, 150 µl medium was added to the cultures of *t* = −4 or *t* = 0 cultures, and 100 µl medium was added to the untreated cultures, which at 4 h p.i. received 50 µl medium supplemented with PEG-IFN to reach a final concentration of 0–1000 ng PEG-IFN ml^−1^. At 48 h p.i., RNA was isolated from 50 µl cell-culture supernatant and quantified using virus-specific real-time RT-PCR assays (see above). At 48 h p.i., CPE was also scored microscopically as either none (0), mild (1), moderate (2), severe (3) or complete (4).

For p-STAT1 nuclear translocation experiments, Vero cells were infected with MERS-CoV or SARS-CoV (m.o.i. of 1). At 8 h p.i., cells were treated with 1000 ng PEG-IFN ml^−1^ for 30 min, fixed with 4 % formaldehyde and 70 % ethanol and subsequently stained for the presence of viral antigen and p-STAT1 translocation.
